# Assessing the neuroendocrine and psychological effects of acute everolimus administration in healthy male participants

**DOI:** 10.1016/j.bbih.2025.101120

**Published:** 2025-10-06

**Authors:** Lucie Jacquet, Anna Lena Friedel, Elisa Orth, Nathalie Reiser, Tina Hörbelt-Grünheidt, Sophie Wiczoreck, Oliver Witzke, Manfred Schedlowski, Marie Jakobs

**Affiliations:** a Institute of Medical Psychology and Behavioral Immunobiology, Center for Translational Neuro- & Behavioral Sciences (C-TNBS), University Medicine Essen, University Duisburg-Essen, Germany; b Department of Infectious Diseases, West German Centre of Infectious Diseases, University Medicine Essen, University Duisburg-Essen, Essen, Germany; c Department of Clinical Neuroscience, Osher Center for Integrative Medicine, Karolinska Institutet, 171 77, Stockholm, Sweden

**Keywords:** Everolimus, Immunosuppression, Adverse side effects, Neuroendocrine, Psychiatric

## Abstract

Previous experimental studies have shown that immunosuppressive mechanistic target of rapamycin inhibitors can induce neuropsychological changes, such as anxiety and depression, in healthy rodents. Furthermore, psychiatric conditions including anxiety have been reported in transplant patients and healthy subjects receiving the mechanistic target of rapamycin inhibitor everolimus. Thus, the present study aimed to further investigate the potentially dose-dependent neuroendocrine and psychological adverse side effects of acute everolimus intake in healthy male subjects. To this end, P70S6 kinase and Akt expression and phosphorylation in peripheral mononuclear blood cells as well as plasma and saliva cortisol, plasma noradrenaline and plasma dehydroepiandrosterone sulfate have been evaluated via western blotting and ELISA. State anxiety and depression have been assessed using questionnaires. Administering 2.5 mg of everolimus four times significantly increased blood peak levels. Additionally, acute everolimus intake led to decreased P70S6 kinase and slightly increased Akt phosphorylation, while protein expression remained unregulated. However, no effects on neuroendocrine parameters including cortisol, noradrenaline and dehydroepiandrosterone sulfate have been reported. Consistent with these findings, acute everolimus administration had no impact on psychological parameters, such as anxiety and depression. Overall, the present study demonstrated that the acute administration of 2.5 mg everolimus in healthy men does not lead to neuroendocrine or psychological adverse side effects. However, as other studies have reported neuroendocrine alterations as well as anxiety- and depression-like symptoms at lower everolimus doses, these findings should be further verified to determine whether everolimus induces psychiatric side effects in a dose-dependent manner.

## Highlights

• Acute everolimus intake had no effect on cortisol, noradrenaline and DHEA-S levels.
• Acute everolimus intake did not induce anxiety- and depression-like symptoms.
• Our results differ from those obtained in studies using lower everolimus doses.
• Everolimus may induce psychiatric side effects in a dose-dependent manner.

## 1 Introduction

Numerous animal studies have reported that immunomodulating treatments not only affect immune responses but also induce neuropsychological changes (Bosche et al., 2015). For example, experimental data demonstrated that immunosuppressive drugs, such as the mechanistic target of rapamycin (mTOR) inhibitor rapamycin, can cause anxiety- and depressive-like behaviors in rodents (Hadamitzky et al., 2014, 2018; Russo et al., 2013; Tsai et al., 2013; Yu et al., 2013). However, other animal studies provide a contrasting perspective, reporting beneficial effects of mTOR inhibitors on neuropsychological impairments (Cambiaghi et al., 2013; Chong et al., 2010; Cleary et al., 2008; Russo et al., 2013). Unfortunately, clinical findings are as inconsistent as those from animal studies, emphasizing the need for further research.

Due to its immunosuppressive and anti-proliferative effects, the mTOR inhibitor and rapamycin analogue everolimus (EVR) is widely used in clinical settings to prevent organ rejection after transplantation (Berger et al., 2019; De Simone et al., 2017; Pascual et al., 2019; Wang and Eisen, 2022; Witzke et al., 2016) and to treat several malignancies including breast cancer, neuroendocrine gastrointestinal tumors, and renal cell carcinoma (Barragan-Carrillo et al., 2024; Blagosklonny, 2023; Ito et al., 2021). However, EVR use is associated with numerous adverse side effects, ranging from infections to stomatitis, glucose or lipid metabolism disorders, and hematopoietic disorders (Arena et al., 2021; Wang and Eisen, 2022). Furthermore, psychiatric conditions such as insomnia and anxiety have been reported in ≥1/10 patients in clinical trials (Eisen et al., 2013; Novartis, 2018) and in healthy subjects (Hörbelt et al., 2020), when 1.5 mg or 2.25 mg EVR were administered. Contrastingly, favorable (Lang et al., 2009) or no effects of EVR (Hörbelt et al., 2020) on affective symptoms and anxiety have also been demonstrated at EVR blood levels of about 5 ng/mL and at doses of 3 mg EVR, suggesting dose-dependency.

On a neuroendocrine basis, stress hormones such as cortisol and noradrenaline are well known to play a significant role in regulating anxiety and depression (Bang et al., 2023; Fiksdal et al., 2019; Qin et al., 2023; Tanaka et al., 2000). However, less attention has been given to sex hormones or their precursors such as dehydroepiandrosterone (DHEA) and its derivate DHEA-sulfate (DHEA-S), despite accumulating evidence showing their association with anxiety and depression (Kurita et al., 2013; Leff-Gelman et al., 2020; Lopresti et al., 2019; Maninger et al., 2009).

Given that EVR is often administered in multimorbid patients in combination with other drugs, such as glucocorticoids or calcineurin inhibitors (De Simone et al., 2017; Pascual et al., 2019), distinguishing its potential neuroendocrine and psychological side effects from those caused by the underlying disease or co-medication remains challenging. Additionally, experimental data on the neuroendocrine and psychological effects of EVR exclusively in healthy subjects is limited, and ethical considerations prevent studies investigating its long-term use in healthy humans.

Contributing to the ongoing discussion about dose-dependency, while excluding clinical confounding variables, the present study aimed to assess the neuroendocrine and psychological effects of acute 2.5 mg EVR administration in healthy men by measuring saliva and plasma cortisol, plasma noradrenaline, and plasma DHEA-S as well as state anxiety and depression. This dose had not been systemically investigated in previous human studies but could provide important clinical insights.

## 2 Materials and methods

### 2.1 Study participants

Healthy male volunteers (*n =* 12) with a mean age of 27.75 ± 1.31 (range 20–37 years), a mean body mass index (BMI) of 24.32 ± 0.62 (range 21.5–26.85) were recruited through public advertisement in the surrounding community. All volunteers underwent an extensive physical and psychiatric assessment, which included self-reported questionnaires and interviews about their medical history as well as a comprehensive blood examination. These evaluations were conducted and subsequently reviewed by physicians of the Department of Infectious Diseases at the University Medicine Essen. Detailed inclusion and exclusion criteria are described in Supplementary Table 1.

Participants were thoroughly informed about the study protocol and gave written informed consent. Privacy rights of human subjects have been observed. The study design was approved by the local ethics committee for human investigations of the University Medicine Essen (17-7500-BO, July 2017) and was conducted according to the principles of the Declaration of Helsinki. Volunteers' sociodemographic, psychological and physiological characteristics are depicted in Table 1.

**Table 1:** Participants' (n = 12) sociodemographic, psychological and physiological characteristics at baseline.

sociodemographic parameters	

age, years	27.75 ± 1.31
BMI, kg/m^2^	24.32 ± 0.62
school education, >12 years	100 %

**psychological parameters**	

trait anxiety	17 ± 1.41
trait depression	16.75 ± 1.44

**physiological parameters**	

blood pressure, mmHG	
systolic	133.75 ± 2.75
diastolic	81.58 ± 2.27
heart rate, beats/min	70.33 ± 3.04
temperature, °C	35.93 ± 0.54

Data are presented as mean ± standard error of mean (SEM) or percentage; BMI, Body Mass Index.

### 2.2 Study design

**Fig. 1:**
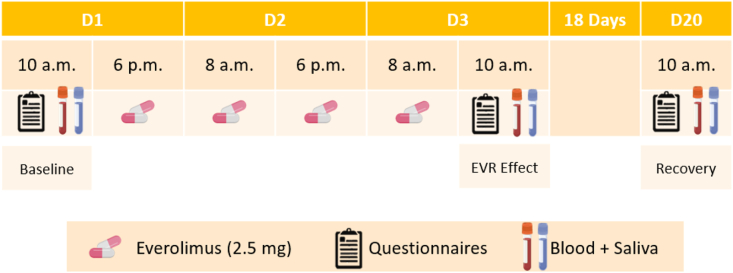
Study design. Study participants received four oral administrations of 2.5 mg EVR every 10–12 h starting on D1 at 6 p.m. To analyze EVR-induced effects, blood and saliva were collected at 10 a.m. on D1 (baseline), D3 (EVR effect) and on D20 (recovery). Additionally, participants filled in questionnaires immediately before sampling. Created with Biorender.

Study participants received four oral administrations of 2.5 mg EVR (Certican; Novartis Pharmaceuticals) every 10–12 h (8 a.m. and 6 p.m.) starting on day (D)1 at 6 p.m. The drug dose was chosen based on a previous exploratory study (Hörbelt et al., 2020). To assess EVR-induced effects, blood and saliva were collected on D1 (baseline), D3 (EVR effect) and on D20 (recovery) at 10 a.m., which was 2 h after EVR administration and corresponded to peak EVR blood levels. Additionally, participants completed questionnaires immediately before sampling session (Fig. 1).

### 2.3 EVR blood level

EVR blood levels on D3 and D20 were determined in ethylenediamine tetra-acetic acid (EDTA) whole blood (Sarstedt) by an external laboratory medicine department using validated liquid chromatography tandem mass spectrometry.

### 2.4 Peripheral blood mononuclear cell isolation

PBMCs were isolated from lithium-heparin whole blood (Sarstedt) using density-gradient centrifugation (Ficoll-Paque Plus; GE Healthcare) and SepMate PBMC Isolation Tubes (STEMMCELL Technologies). PBMCs were washed in protein-free phosphate-buffered saline (PBS) and adjusted to 10^6^ cells/ml in cell culture medium (RPMI 1640; Life Technologies) using a fully automatic 3-part cell counter (Sysmex).

### 2.5 Western blotting

Isolated PBMCs were incubated at 37 °C in 5 % CO_2_ for 4 h. To analyze Akt protein expression and activity, PBMCs were stimulated with 50 ng/ml phorbol 12-myristate 13-acetate (PMA; Sigma-Aldrich) the last 15 min. Afterwards, PBMCs were washed in ice-cold PBS and lysed in radioimmunoprecipitation assay (RIPA) buffer (RIPA Lysis Buffer System; Santa Cruz Technologies) for 10 min on ice with repeated vortexing. After centrifugation (10 min, 14.000×*g*, 4 °C), liquid protein was collected. Protein concentrations were measured using a Pierce bicinchoninic acid assay (BCA) Protein Assay (ThermoFisher Scientific). For P70S6K and Akt protein analyses, 30 μg protein were mixed with 1x protein gel loading buffer (Roti®Load 1, CARL ROTH), heated for 5 min at 95 °C and separated using 10 % sodium dodecyl sulfate polyacrylamide (SDS-PAGE) gels and SDS-PAGE electrophoresis. Then, proteins were transferred to polyvinylidene fluoride (PVDF) membranes (Trans-Blot Turbo Transfer System; BIO-RAD) and stained with pP70S6K-Thr389 (1:1000; Cell Signaling, #9205), P70S6K (1:1000; Cell Signaling, #5707), pAkt-Ser473 (1:1000; Cell Signaling, #9271) and Akt (1:1000; Cell Signaling, #9272) primary antibodies overnight. Glyceraldehyde 3-phosphate dehydrogenase (GAPDH, 1:1000; Cell Signaling, #2118) antibody was used as a loading control. The next day, membranes were washed and incubated with horseradish peroxidase (HRP)-conjugated secondary anti-rabbit (1:1000; Cell Signaling, #7074) antibody for 1 h. Finally, target proteins were visualized by chemiluminescence using Immobilon Western HRP Substrate detection reagents (Merck Millipore) and a Fusion Imager (PEQLAB Biotechnology). Protein signals were quantified using ImageJ.

### 2.6 Neuroendocrine parameter

By centrifugation, plasma (10 min, 2000×*g*, 4 °C) was collected from EDTA whole blood and saliva (2 min, 1000×*g*, 4 °C) was collected from Salivettes (Sarstedt). To measure saliva cortisol (IBL International), plasma cortisol (IBL International), plasma noradrenaline (LDN) and plasma DHEA-S (ThermoFisher Scientific) concentrations, commercial enzyme linked immunosorbent assays (ELISA) with a sensitivity of 0.15 ng/ml, 20 ng/ml, 36 pg/ml and 90.9 pg/ml were used according to manufacturers' instructions. Optical density was determined on a Fluostar OPTIMA Microplate Reader (BMG Labtech) set to 450 nm. Absolute hormone concentrations were calculated using a log–log curve-fit standard curve.

### 2.7 Questionnaires

Participants' sociodemographic data were collected prior to D1. Anxiety and depression were measured using the State-Trait Anxiety and Depression Inventory (STADI), a validated questionnaire measuring anxiety and depression as a state and a trait with two self-report questionnaires of 20 items each, answerable on a Likert scale from 1 (‘almost never’/‘not at all’) to 4 (‘almost always’/‘very much’) (Laux et al., 2013). Trait anxiety and trait depression were measured on D1, while state anxiety and state depression were measured immediately before blood sampling on D1, D3 and D20. Scores can range from 10 to 40, with higher scores indicating higher levels of anxiety or depression.

### 2.8 Statistical analysis

Statistical analyses were performed using PASW statistics (SPSS, version 30) and GraphPad Prism (Version 9). The assumption of normality was examined using the Shapiro-Wilk test, since this test has a high statistical power, especially for sample sizes *<* n = 50 (Razali, 2011). As many parameters did not meet the normality assumption and sample sizes were relatively small, non-parametric calculations were conducted, with time point of measurement as independent variable and all biological, physiological and psychological parameters as dependent variables. To analyze changes over time, Friedman-ANOVA was used, respectively Wilcoxon-test for EVR-levels, as only two time points are available here. When Friedman-ANOVA was significant, individual differences between time points were assessed applying Dunn's post-hoc multiple comparison test. Bonferroni correction was applied for multiple comparisons. The level of significance was set at *p* < 0.05 (for multiple comparisons *p* < 0.05/number of comparisons). *χ*^*2*^-statistics are reported for Friedman-ANOVA and Z-statistics for Wilcoxon and Dunn's test. In addition, effect sizes are provided as Kendall's W (for Friedman ANOVA) and Pearson's r (for Wilcoxon-test/Dunn's test). Results are presented as mean ± SEM.

## 3 Results

### 3.1 EVR blood levels

Since EVR intake within the last 30 days prior to the start of the study was an exclusion criterion, no circulating EVR was expected on D1 (baseline). Therefore, EVR blood levels were measured on D3 after acute EVR administration and on D20 (recovery) for a subset of participants (n = 6) as previous studies have already demonstrated increased EVR blood levels following acute intake (Hörbelt et al., 2020). Our data confirmed that EVR peak blood levels were significantly elevated on D3 compared to D20 (Z = −2.201, *p* = 0.031, r = −0.635), when blood levels returned to (nearly) 0 ng/ml.

### 3.2 Effect of EVR on mTOR-regulated protein activity

To evaluate the efficacy of acute EVR administration, mTOR-regulated P70S6K and Akt protein activity/phosphorylation (pP70S6K-Thr389, pAkt-Ser473) have been analyzed in PBMCs via western blotting.

**Fig. 2:**
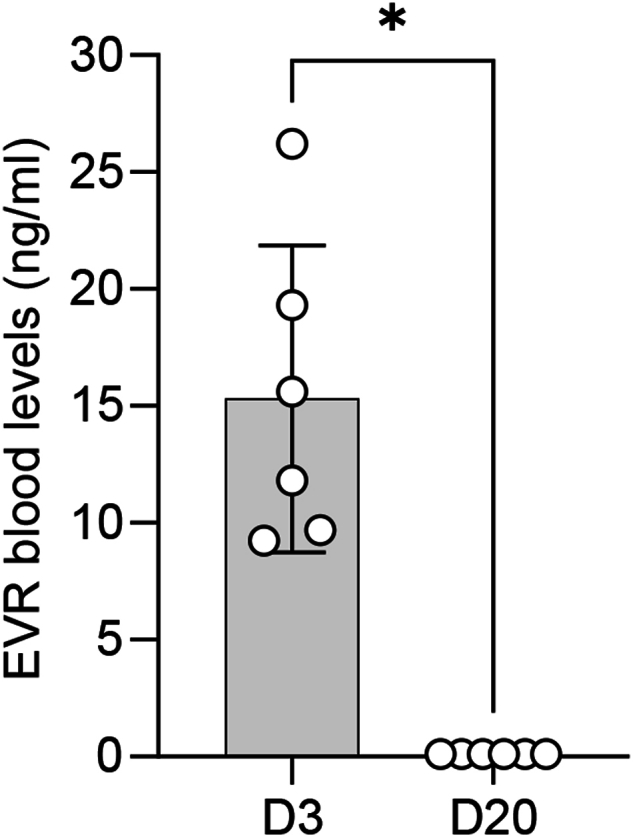
EVR blood levels after acute EVR intake On D3, after 2.5 mg EVR has been orally administered four times every 10–12 h, EVR blood levels were significantly increased. In contrast, on D20 (recovery) no circulating EVR has been detected. Asteriks indicate a statistically significant difference between time points (Wilcoxon-test; ∗*p* < 0.05; n = 6). Results are presented as mean ± SEM.

Acute EVR intake resulted in a significant reduction of pP70S6K on D3 compared to D1 (Z = 2.981, *p* < 0.01, r = 0.609). However, P70S6K activity recovered on D20 and no longer differed significantly from baseline levels (Z = 0.622, *p* = 0.534, r = 0.133) (Fig. 2A). In contrast, P70S6K protein expression was not affected by acute EVR administration (D3) and the recovery interval (D20) (Fig. 2C; χ^2^ (2) = 2, *p* = 0.368, W = 0.083).

Although the increase was not statistically significant (χ^2^ (2) = 3.167, *p* = 0.205, W = 0.132), pAkt slightly increased throughout the study, reaching a maximum of 137 % compared to baseline (D1) on D20 (Fig. 2D). Similar to P70S6K, Akt protein expression remained unaffected over time (Fig. 2E; χ^2^ (2) = 1.5, *p* = 0.472, W = 0.063).

### 3.3 Effect of EVR on neuroendocrine parameters

Neuroendocrine hormone analyses revealed that acute EVR intake (D3) and the recovery interval (D20) had no effect on saliva cortisol (Fig. 4A; χ^2^ (2) = 0.5, *p* = 0.779, W = 0.021), plasma cortisol (Fig. 4B; χ^2^ (2) = 1.167, *p* = 0.558, W = 0.049), plasma noradrenaline (Fig. 4C; χ^2^ (2) = 3.5, *p* = 0.174, W = 0.146) or plasma DHEA-S (Fig. 4D; χ^2^ (2) = 1.5, *p* = 0.472, W = 0.063) secretion over time.

### 3.4 Vital parameters

Consistence with the neuroendocrine data, no effects of acute EVR administration (D3) and the recovery interval (D20) were observed on systolic (χ^2^ (2) = 2.085, *p* = 0.353, W = 0.087), diastolic blood pressure (χ^2^ (2) = 2.851, *p* = 0.240, W = 0.119), heart rate (χ^2^ (2) = 0.311, *p* = 0.856, W = 0.013) and temperature (χ^2^ (2) = 2.596, *p* = 0.273, W = 0.108) over time (Supplementary Table 2).

### 3.5 Effect of EVR on anxiety and depression

To assess the effect of acute EVR administration (D3) and the recovery interval (D20) on state anxiety and depression, psychological questionnaires were completed. The results revealed no significant impact on state anxiety (Fig. 5A; χ^2^ (2) = 0.889, *p* = 0.667, W = 0.040) or depression (Fig. 5B; χ^2^ (2) = 3.765, *p* = 0.148, W = 0.171) over time.

## 4 Discussion

**Fig. 3:**
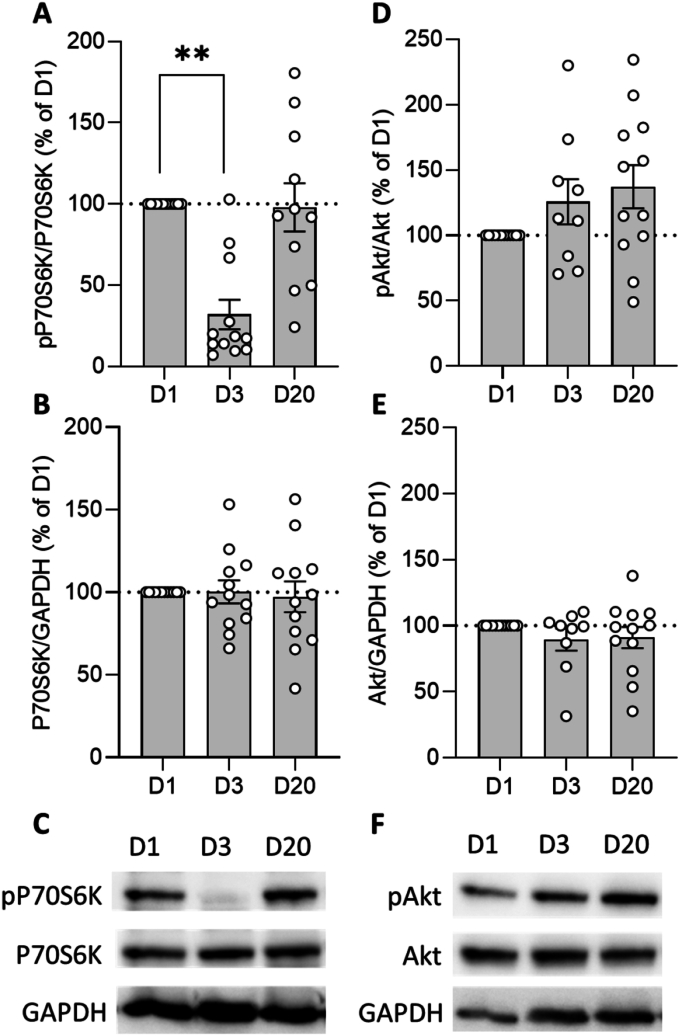
Effect of acute EVR intake on P70S6K and Akt protein expression and activity. Acute oral EVR administration (four times, every 10–12 h) resulted in a significantly reduced P70S6K activity/phosphorylation on D3, while P70S6K activity recovered on D20 (A). In contrast, Akt activity/phosphorylation increased throughout the study (D). Further, Western blot analyses revealed that neither P70S6K (B) nor Akt (E) protein expression were affected by acute EVR intake. Results are also depicted by representative blots for P70S6K (C) and Akt (F). Asterisks indicate statistically significant differences between time points (Friedman test, Dunn's multiple comparison test; ∗∗*p* < 0.01; n = 9–12). Results are presented as mean percentage changes normalized to baseline (D1) ± SEM.

**Fig. 4:**
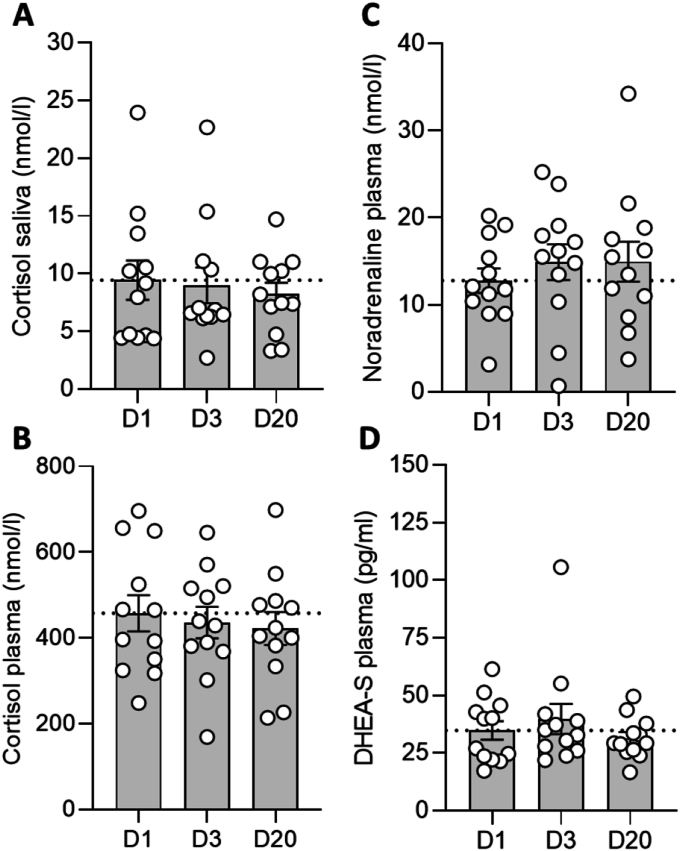
Effect of acute EVR intake on neuroendocrine hormone secretion Acute oral EVR administration (D3, four times, every 10–12 h) did not affect saliva cortisol (A), plasma cortisol (B), plasma noradrenaline (C) or plasma DHEA-S (D) secretion compared to baseline levels (D1). Results are presented as mean ± SEM (n = 12).

**Fig. 5:**
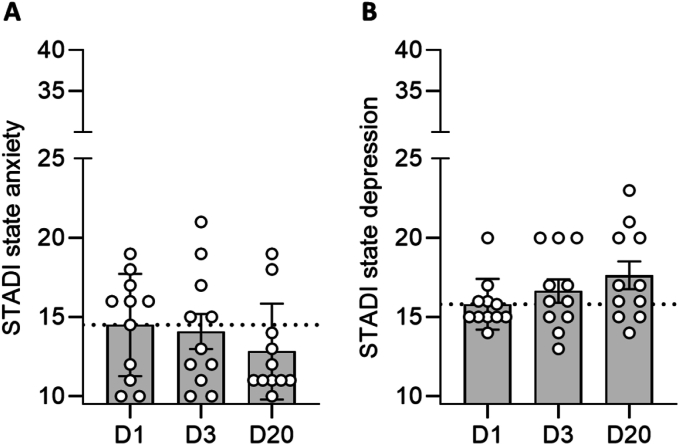
Effect of acute EVR intake on anxiety and depression Acute oral EVR administration (D3, four times, every 10–12 h) did not affect state anxiety (A) or depression (B) compared to baseline levels (D1). Results are presented as mean ± SEM (n = 10–11).

The present study aimed to investigate the potentially dose-dependent neuroendocrine and psychological adverse side effects of acute EVR intake in healthy male subjects. Administering 2.5 mg of the immunosuppressive mTOR inhibitor EVR four times significantly increased blood peak levels (Fig. 2). Additionally, acute EVR intake led to decreased P70S6K and slightly increased, but non-significant, Akt protein phosphorylation/activity, while protein expression remained unregulated (Fig. 3). However, no effects on neuroendocrine parameters such as saliva cortisol, plasma cortisol, plasma noradrenaline and plasma DHEA-S have been reported (Fig. 4). Consistent with these findings, acute EVR administration had no impact on psychological parameters, including anxiety and depression (Fig. 5).

Previous experimental studies have shown that the mTOR inhibitor, and EVR analogue rapamycin can induce neuropsychological changes, such as anxiety and depression, in healthy rodents (Bosche et al., 2015; Hadamitzky et al., 2014, 2018; Russo et al., 2013; Tsai et al., 2013; Yu et al., 2013). Furthermore, psychiatric conditions such as anxiety have been reported in transplant patients (Novartis, 2018) and healthy subjects (Hörbelt et al., 2020) receiving EVR treatment. In these clinical trials, psychiatric adverse side effects were observed following 1.5 mg EVR intake (Novartis, 2018). Similarly, in the preliminary study with healthy subjects, an anxiety-inducing effect has been detected at doses of 1.5 mg or 2.25 mg, but not at 3 mg EVR (Hörbelt et al., 2020). Thus, the present finding that 2.5 mg EVR did not affect state anxiety and depression partially aligns with these observations, suggesting that the depression- and anxiety-inducing properties of EVR may only occur at low- or medium-doses, while the 2.5 mg dose might represent the upper threshold for such effects. However, given the limited data on neuropsychological changes induced by EVR in healthy individuals, these results require further verification. Future experiments should aim to disentangle psychiatric effects directly associated with EVR from those related to comorbidities or co-medication.

In line with the psychological results, no effect of 2.5 mg EVR on neuroendocrine parameters had been reported in the present study. Given that increases in stress hormones such as cortisol and noradrenaline are positively associated with anxiety and depression (Bang et al., 2023; Chan and Wu, 2024; Fiksdal et al., 2019; Qin et al., 2023; Tanaka et al., 2000), whereas decreases in DHEA-S levels correlate with depressive and anxious symptomatology (Nenezic et al., 2023), these negative findings support the hypothesis that acute intake of 2.5 mg EVR does not induce psychiatric adverse side effects. Notably, a previous study observed a rise in plasma noradrenaline with 1.5 mg or 2.25 mg EVR, whereas a significant decrease in plasma noradrenaline, plasma cortisol as well as saliva cortisol was reported with 3 mg EVR (Hörbelt et al., 2020). These findings suggest a potentially dose-dependent effect of EVR, but further validation is required.

A possible explanation for our observations might be a non-monotonic dose-response (NMDR) relationship. NMDRs have been described across pharmacology and toxicology, including U-shaped profiles in which the strongest responses are observed at both low and high exposure levels, with intermediate doses producing attenuated or absent effects. These unconventional responses can arise from numerous molecular mechanisms such as opposing effects induced by multiple receptors differing by their affinity, receptor desensitization, negative feedback with increasing dose, or dose-dependent metabolism modulation (Hill et al., 2018; Lagarde et al., 2015; Zuardi et al., 2017).

The brain-derived neurotrophic factor (BDNF) has been shown to play a critical role in the pathogenesis of depression and anxiety (Colucci-D'Amato et al., 2020; Meng et al., 2021; Peters et al., 2020). Since BDNF acts as an upstream regulator of Akt and promotes the activation of its signaling pathway, Akt has also been implicated in psychiatric disorders (Jin et al., 2022; Liu et al., 2022; Wu et al., 2018; Zhou et al., 2014). Indeed, several rodent studies have demonstrated that antidepressant effects positively correlate with increased or restored pAkt expression (Carrier et al., 2015; Guo et al., 2024; Qin et al., 2024). Similarly, anxiety-related behaviors have been linked to the downregulation of BDNF and pAkt in female mice (Meng et al., 2021). Consistent with this, patients with major depressive disorders exhibit significantly reduced pAkt expression in the hypothalamus (Li et al., 2016).

Although not statistically significant, the present study observed a slight increase in pAkt expression, further supporting the hypothesis that the chosen EVR dose did not impair the Akt pathway and therefore, did not induce anxiety- or depression-like symptoms. In general, an upregulation of pAkt can result from the inhibitory effect of EVR on the mTOR complex 1 (mTORC1), as reflected by a significantly diminished pP70S6K expression. By inhibiting mTORC1, the negative feedback loop that restricts pAkt expression is also blocked (Chen et al., 2013; Panwar et al., 2023). Comparable protein expression analyses for lower EVR doses that induced anxiety- and depression-like symptoms (Hörbelt et al., 2020) are currently unavailable but could help to elucidate potential underlying mechanisms.

However, some limitations should be considered when interpreting our results. First, women have a 2-fold higher risk of developing an anxiety or depressive disorder compared to men (Fracas et al., 2023; Kessler et al., 2003). Therefore, to assess the risk of EVR in inducing anxiety- or depression-like symptoms, further experiments including female participants are necessary. Due to the exploratory character of this study and the relatively small sample size, we chose to first focus on males, since women show different stress hormone responses, dependent on their menstrual cycle phase (Dearing et al., 2022; Hellhammer et al., 2009; Kajantie and Phillips, 2006; Kirschbaum et al., 1999).

Second, we chose a within-subject repeated-measures study design to minimize inter-individual variability (“random noise”), especially in neuroendocrine and psychological outcomes, which are known to fluctuate across individuals (Hellhammer et al., 2009; Zänkert et al., 2019). However, including a control group would strengthen the robustness of the findings.

Moreover, we analyzed pP70S6K and pAkt expression only in PBMCs. While this provides valuable peripheral data, central pAkt expression would be more meaningful in the context of psychiatric disorders. However, central pAkt cannot be analyzed in living humans. Nevertheless, an animal study has demonstrated that peripherally administered rapamycin also downregulates the expression of pP70S6K in the amygdala, a key brain region involved in emotional regulation (Lückemann et al., 2021).

Overall, the present study demonstrated that the acute administration of 2.5 mg EVR in healthy men does not lead to neuroendocrine or psychological adverse side effects. However, as other studies have reported neuroendocrine alterations as well as anxiety- and depression-like symptoms at lower EVR doses (Hörbelt et al., 2020; Novartis, 2018), these findings should be further verified to determine whether EVR induces psychiatric side effects in a dose-dependent manner.

From a clinical perspective, this information is particularly important for the treatment of transplant patients, as the general dosage recommendation (adjusted to individual trough levels) typically ranges from 1 to 3 mg EVR per day (Novartis, 2018). Considering that EVR is administered on a daily basis in clinical contexts, even small dose adjustments and the knowledge about their neuroendocrine and psychiatric effects might contribute to the patients' well-being.

## CRediT authorship contribution statement

**Lucie Jacquet:** Writing – original draft, Methodology, Investigation, Data curation, Conceptualization. **Anna Lena Friedel:** Writing – review & editing, Formal analysis, Data curation, Conceptualization. **Elisa Orth:** Methodology, Investigation. **Nathalie Reiser:** Methodology, Investigation. **Tina Hörbelt-Grünheidt:** Conceptualization. **Sophie Wiczoreck:** Methodology, Investigation. **Oliver Witzke:** Funding acquisition, Conceptualization. **Manfred Schedlowski:** Funding acquisition, Conceptualization. **Marie Jakobs:** Writing – review & editing, Writing – original draft, Visualization, Supervision, Methodology, Investigation, Formal analysis, Data curation, Conceptualization.

## Funding

This research did not receive any specific grant from funding agencies in the public, commercial, or not-for-profit sectors.

## Declaration of competing interest

The authors declare the following financial interests/personal relationships which may be considered as potential competing interests: Oliver Witzke reports a relationship with Amgen, Alexion, Astellas, AstraZeneca, Basilea, Biotest, Bristol-Myers Squibb, Correvio, Chiesi, Gilead, GSK, Hexal, Janssen, Dr. F. Köhler 10.13039/501100024870Chemie, 10.13039/100030732MSD, 10.13039/100004336Novartis, 10.13039/100004337Roche, 10.13039/100004319Pfizer, 10.13039/100004339Sanofi, TEVA and UCB that includes: funding grants, speaking and lecture fees, and travel reimbursement. If there are other authors, they declare that they have no known competing financial interests or personal relationships that could have appeared to influence the work reported in this paper.

## Data Availability

Source data for this study are openly available at Open Science Framework (DOI 10.17605/OSF.IO/GYNQ3).
