# Supplementary material for: Assessing the neuroendocrine and psychological effects of acute everolimus administration in healthy male participants
Source: Brain Behav Immun Health. 2025 Oct 6;49:101120. doi: 10.1016/j.bbih.2025.101120 (PMC12547271; doi:10.1016/j.bbih.2025.101120)
Supplement: Multimedia component 1 [file mmc1.pdf]

Supplementary material

Inclusion and exclusion criteria

Study inclusion and exclusion criteria are summarized in Supplementary Table 1. Twelve healthy men were included. Nine men were excluded before study start.

Supplementary Table 1: Inclusion and exclusion criteria

Inclusion criteria	***Exclusion criteria***
1. Healthy male subjects 18-40 years of age	1. Everolimus intake in the last 30 days prior to the first administration of the study drug
2. Body mass Index (BMI) between 18.5kg/m2 and 30.0kg/m2	2. Acute and chronic infectious diseases
3. Weight over 55kg	3. History of a relevant manifest disease of the hepatobiliary system, kidneys, nervous system, immune system, respiratory system, urinary system, digestive system, endocrine system, blood/tumor, cardiovascular system and mental illnesses
4. Vital parameters: Heart rate between 40-100/min, blood pressure <140/90mmHg, temperature <38.5°C	4. Regular use of medication
5. Extract from the laboratory tests requirements: c-reactive protein (CRP) <2mg/l, creatinine kinase (CK) <250U/l, cholesterol (total) <239mg/dl, low density lipoprotein (LDL) <115mg/dl, bilirubin (total) <1.2mg/dl, creatinine <1.3mg/dl, hemoglobin A1c (HbA1c) <5.7%, normal blood count	5. Medication intake which induces or inhibits metabolizing enzymes within 30 days prior to the first administration of the study drug
6. Electrocardiogram (ECG): QTc according to Bazett <450ms	6. Administration of vaccinations within 30 days prior to the first administration of the study drug
	7. Hypersensitivity to everolimus, other rapamycin derivatives or other components of the study drug
	8. Nicotine abuse, signs of drug or alcohol abuse

Effect of EVR on participants´ vital parameters

No effects of acute EVR administration (D3) on systolic (p = 0.353), diastolic blood pressure (p = 0.240), heart rate (p = 0.856) and temperature (p = 0.273) have been reported.

Supplementary Table 2: Participants´ (n = 11-12) vital parameters throughout the study.

	**D1**	**D3**	**D20**
blood pressure, mmHG			
systolic	133.75 ± 2.75	136.09 ± 3.99	132.33 ± 2.74
diastolic	81.58 ± 2.27	83.82 ± 3.38	81.42 ± 2.25
heart rate, beats/min	70.33 ± 3.04	72.73 ± 3.88	68.58 ± 2.91
temperature, °C	35.93 ± 0.54	35.77 ± 0.19	35.87 ± 0.15

Data are presented as mean ± SEM.
